# Chemogenetic dissection of a prefrontal-hypothalamic circuit for socially subjective reward valuation in macaques

**DOI:** 10.1038/s41467-023-40143-x

**Published:** 2023-07-20

**Authors:** Atsushi Noritake, Taihei Ninomiya, Kenta Kobayashi, Masaki Isoda

**Affiliations:** 1 Division of Behavioral Development, Department of System Neuroscience, National Institute for Physiological Sciences, National Institutes of Natural Sciences, Okazaki, Japan; 2 Department of Physiological Sciences, School of Life Science, The Graduate University for Advanced Studies (SOKENDAI), Hayama, Japan; 3 Section of Viral Vector Development, National Institute for Physiological Sciences, National Institutes of Natural Sciences, Okazaki, Japan

**Keywords:** Social behaviour, Motivation, Reward, Neurophysiology

## Abstract

The value of one’s own reward is affected by the reward of others, serving as a source for envy. However, it is not known which neural circuits mediate such socially subjective value modulation. Here, we chemogenetically dissected the circuit from the medial prefrontal cortex (MPFC) to the lateral hypothalamus (LH) while male macaques were presented with visual stimuli that concurrently signaled the prospects of one’s own and others’ rewards. We found that functional disconnection between the MPFC and LH rendered animals significantly less susceptible to others’ but not one’s own reward prospects. In parallel with this behavioral change, inter-areal coordination, as indexed by coherence and Granger causality, decreased primarily in the delta and theta bands. These findings demonstrate that the MPFC-to-LH circuit plays a crucial role in carrying information about upcoming other-rewards for subjective reward valuation in social contexts.

## Abstract (web summary)

How subjective reward value is affected by social comparison remains poorly understood. Here, the authors show a crucial role for the circuit from the medial prefrontal cortex to the lateral hypothalamus in subjective value modulation in social contexts.

## Introduction

Reward affects motivational processes. This is true for rewards that are directly experienced as well as those that are observed, i.e., others’ rewards. For example, the value of one’s own rewards is not determined based solely on their absolute amount, but is often affected by the amount of others’ rewards, serving as a source for envy. Reward valuation is a subjective process based on comparisons between self and others^1^. Human neuroimaging has shown that the medial prefrontal cortex (MPFC) and subcortical nuclei are involved in social reward valuation and related decision-making^2–4^. Rewards for the self and others are encoded by distinct neurons in the MPFC and lateral hypothalamus (LH), while subjective reward value is encoded by neurons in the LH and dopaminergic (DA) midbrain nuclei in macaques^5,6^. These findings suggest that agent-specific reward information is integrated to encode a subjective value through the circuits from the MPFC to subcortical nuclei. However, this has not been tested using intervention methodology.

In the present study, we performed circuit-selective blockade using dual viral vector infection in macaques. We selected the circuit from the MPFC to the LH because of three reasons. First, LH has long been considered a subcortical node in the reward system^7^. Second, LH and adjacent structures are increasingly recognized as part of social brain networks in rodents^8^, macaques^9^, and humans^10^. Lastly, electrophysiological recordings in macaques have demonstrated prominent information flow from the MPFC to the LH during subjective reward valuation in social contexts^6^. Here, we show that monkeys with functional disconnection between the MPFC and LH were significantly less influenced by others’ reward prospects, while they remained sensitive to their own reward prospects.

## Results

**Fig. 1:**
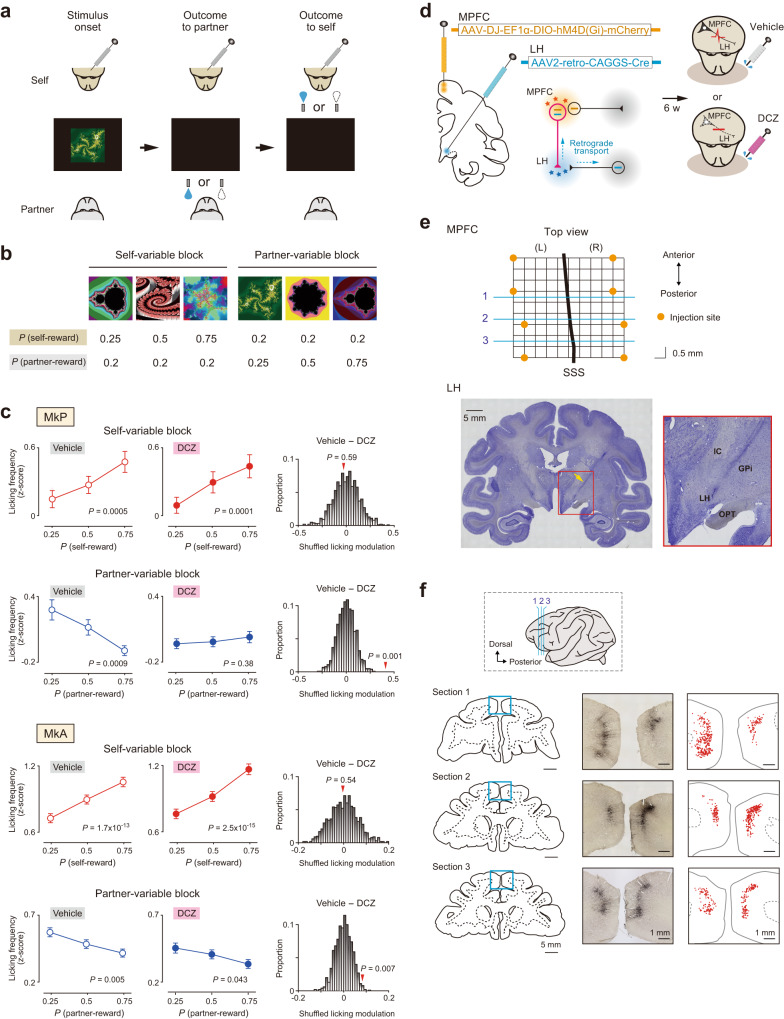
Selective blockade of MPFC-to-LH circuit diminished susceptibility to others’ reward. **a** Social Pavlovian conditioning procedure. **b** Two sets of three conditioned stimuli. Each stimulus was associated with self-reward and partner-reward probabilities. **c** Correlations between licking frequency and variable-reward probability in vehicle (left) and DCZ (middle) conditions. Licking frequency is *z*-score normalized. Data are mean ± SEM. *P* values from two-sided Spearman’s correlation test (total *n* = 1300, 1229, 1267, and 1229 trials in the vehicle-self, DCZ-self, vehicle-partner, and DCZ-partner condition, respectively, in MkP; total *n* = 3834, 3705, 3594, and 3408 trials in the vehicle-self, DCZ-self, vehicle-partner, and DCZ-partner condition, respectively, in MkA). Monte Carlo permutation results (right). Bars indicate differences in shuffled licking modulation between vehicle and DCZ conditions (*n* = 1000). Red arrowheads indicate actual differences. **d** Dual viral vector infection. Vector constructs for circuit-selective blocking (left). Alternation of vehicle and DCZ experiments (right). **e** Spatial coordinates of injection sites in the MPFC in MkA (top). Blue lines correspond to those in Fig. 1f, inset. Orange circles indicate injection coordinates viewed from top. Note that, in both monkeys, injections were made at eight sites in each hemisphere (i.e., two different depths at four different rostro-caudal coordinates; see Methods). SSS, superior sagittal sinus. The most caudal injection sites were located 4.5 mm anteriorly to the physiological border between the supplementary motor area (SMA) and pre-SMA. Nissl-stained section for injection sites in the LH in MkA (bottom). Yellow arrow indicates gliosis caused by injectrode penetrations (left). Scale bar, 5 mm. Enlarged view of the area indicated by the red rectangle (right). Note that, in both monkeys, injections were made at four sites in each hemisphere (i.e., two different depths along each penetration track at two different rostro-caudal coordinates; see Methods). IC, internal capsule. OPT, optic tract. GPi, internal segment of globus pallidus. **f** Distribution of dual-infected MPFC neurons in MkA. Coronal sections cut through anteroposterior levels shown in inset (left). Anti-mCherry immunohistochemistry for cortical areas indicated by blue rectangles (middle). Locations of mCherry-positive neurons (right). Source data are provided as a Source Data file.

We devised a social Pavlovian conditioning procedure in which aspects of socially subjective reward valuation were incorporated^5^. In each trial (Fig. 1a), two monkeys (*Macaca fuscata*) facing each other were presented with a conditioned visual stimulus, followed by reward feedback (water or nothing) given first to the ‘partner’ and then to the subject monkey (MkP or MkA, designated as ‘self’). Two sets of three conditioned stimuli were used in two trial blocks. Each stimulus was associated with self-reward and partner-reward probabilities (Fig. 1b). In the self-variable block (Fig. 1b, left), the probability of self-reward differed depending on which stimulus was presented (*P* = 0.25, 0.5, or 0.75), while the probability of partner-reward was invariable (*P* = 0.2). In the partner-variable block (Fig. 1b, right), the probability of partner-reward was different (*P* = 0.25, 0.5, or 0.75), while the probability of self-reward was invariable (*P* = 0.2). These two blocks were alternated during data collection.

Using this procedure, we previously reported that licking movement in response to stimulus reflected a subjective, not objective, value of upcoming self-reward. Specifically, the magnitude of licking movement increased as a function of self-reward probability and, importantly, decreased as a function of partner-reward probability despite an objectively constant self-reward probability^5^. The licking modulation in the partner-variable block was socially selective, because it did not develop in a non-social condition in which the partner was physically absent and instead a water-collecting bottle was placed in an empty chair^5^. Therefore, our social Pavlovian conditioning procedure can assess socially subjective reward valuation under the laboratory-based condition.

We performed dual viral vector infection to express an inhibitory designer receptor exclusively activated by designer drug (DREADD)^11^ in target neurons. A retrograde gene transfer vector, AAV2-retro-CAGGS-Cre, was injected into the LH, and an anterograde gene transfer vector, AAV-DJ-EF1α-DIO-hM4D(Gi)-mCherry, was injected into the MPFC (Fig. 1d, e). Using this protocol, only MPFC neurons whose axons terminate in the LH are dually infected, and their spiking activities are silenced with systemic injections of deschloroclozapine (DCZ), a highly selective, potent, and metabolically stable DREADD agonist^12^ (Fig. 1d, right). The injection sites were determined electrophysiologically in both hemispheres (Methods). Postmortem anti-mCherry immunohistochemistry in MkA confirmed that dual-infected neurons were distributed in the dorsomedial convexity of bilateral MPFC (Fig. 1f).

Six weeks after the vector injections, we assessed the impacts of circuit-specific silencing. We alternately performed control and test experiments on separate days. In the vehicle control condition, saline (subject MkP) or dimethyl sulfoxide (DMSO) diluted with saline (subject MkA) was administered intramuscularly. In the test condition, DCZ (0.1 mg/kg) dissolved in DMSO with saline was administered intramuscularly. In both conditions, behavioral data collection started 10–15 min after vehicle or DCZ injections^12,13^. In the vehicle control condition, we replicated previous findings that the licking magnitude increased as a function of self-reward probability and decreased as a function of partner-reward probability (Fig. 1c, left column; MkP, self-variable block, *ρ* = 0.10, *P* = 4.8 × 10^−4^; MkP, partner-variable block, *ρ* = –0.09, *P* = 9.2 × 10^−4^; MkA, self-variable block, *ρ* = 0.12, *P* = 1.7 × 10^−13^; MkA, partner-variable block, *ρ* = –0.05, *P* = 0.005; Spearman’s correlation test). Thus, vehicle injections alone had a negligible, if any, impact on social valuation behavior.

Notably, DCZ injections rendered the monkeys considerably less susceptible to partner’s, but not one’s own, reward. In MkP, the licking magnitude was no longer affected by partner-reward probabilities (Fig. 1c, middle column, second top panel; *ρ* = 0.02, *P* = 0.38; Spearman’s correlation test). In the partner-variable block, differences in the licking movement between the preferred and non-preferred trials (‘licking modulation’; Methods) were significantly smaller in the DCZ than the vehicle conditions, as assessed by the Welch’s *t*-test (*P* = 0.0025) and Monte Carlo permutation test (Fig. 1c, right column, second top panel; *P* = 0.001). In MkA, correlations between licking magnitude and partner-reward probability remained significant after DCZ injections (Fig. 1c, middle column, bottom panel; *ρ* = –0.03, *P* = 0.043; Spearman’s correlation test). However, the licking modulation was significantly smaller in the DCZ than the vehicle condition, as evaluated by the Welch’s *t*-test (*P* = 0.015) and Monte Carlo permutation test (Fig. 1c, right column, bottom panel; *P* = 0.007).

As opposed to the partner-variable block, the licking movement in the self-variable block was largely unaffected. Specifically, correlations between licking magnitude and self-reward probability remained significant in the DCZ condition (MkP, *ρ* = 0.11, *P* = 9.9 × 10^−5^; MkA, *ρ* = 0.13, *P* = 2.5 × 10^−15^; Spearman’s correlation test). Moreover, the licking modulation was not significantly changed by DCZ injections (MkP, *P* = 0.88; MkA, *P* = 0.95; Welch’s *t*-test; MkP, *P* = 0.59; MkA, *P* = 0.54; Monte Carlo permutation test). Thus, the effect of chemogenetic silencing was specific to the processing of partner-reward information, ruling out the possibility that the observed effect was caused by general impairments of stimulus discrimination. These findings establish a crucial role for the top-down signaling from the MPFC to LH in subjective reward valuation by taking others’ reward information into account.

**Fig. 2:**
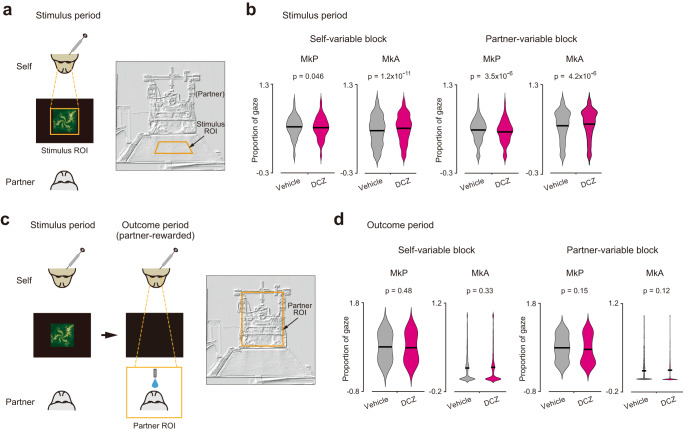
Gaze behavior during stimulus and outcome periods. **a** Stimulus region of interest (ROI) for quantitative analysis. Top view (left) and front view (right). **b** Violin plots showing proportions of time spent looking at the stimulus ROI during the stimulus period. *P* values from two-sided Welch’s *t*-test. *n* = 1511, 1229, 3839, and 3720 trials for the vehicle-MkP, DCZ-MkP, vehicle-MkA, and DCZ-MkA condition, respectively, in the self-variable block. *n* = 1468, 1230, 3599, and 3479 trials for the vehicle-MkP, DCZ-MkP, vehicle-MkA, and DCZ-MkA condition, respectively, in the partner-variable block. Horizontal black lines denote mean values. **c** Partner ROI for quantitative analysis. Top view (left) and front view (right). **d** Violin plots showing proportions of time spent looking at the Partner ROI during the outcome period. Data derived from trials in which Partner was rewarded. *P* values from two-sided Welch’s *t*-test. *n* = 294, 256, 768, and 744 trials for the vehicle-MkP, DCZ-MkP, vehicle-MkA, and DCZ-MkA condition, respectively, in the self-variable block. *n* = 732, 579, 1800, and 1739 trials for the vehicle-MkP, DCZ-MkP, vehicle-MkA, and DCZ-MkA condition, respectively, in the partner-variable block. Horizontal black lines denote mean values. Source data are provided as a Source Data file.

The intervention effect in the partner-variable block was probably not caused by attentional factors. During the stimulus period (Fig. 2a), the monkeys spontaneously looked at the conditioned stimulus almost equally long between the vehicle and DCZ conditions. Although differences in the gaze time between the two conditions were statistically significant (Fig. 2b, right; MkP, *P* = 3.5 × 10^−6^; MkA, *P* = 4.2 × 10^−6^; Welch’s *t*-test), the direction of change was not consistent between the two monkeys. Specifically, MkP exhibited longer gaze durations in the vehicle condition than in the DCZ condition, while MkA exhibited longer gaze durations in the DCZ condition than in the vehicle condition. Furthermore, during the outcome period in which the partner was rewarded (Fig. 2c), the time spent looking at the partner was not significantly affected by DCZ administrations in either monkey (Fig. 2d, right; MkP, *P* = 0.15; MkA, *P* = 0.12; Welch’s *t*-test). Note that gaze behavior was qualitatively similar between the self-variable and partner-variable blocks in each monkey.

**Fig. 3:**
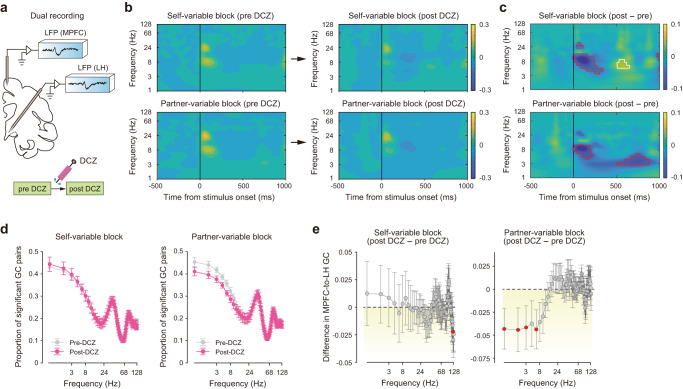
Selective blockade of MPFC-to-LH circuit affected inter-areal coordination. **a** Experimental setup for simultaneous neural recordings. LFP, local field potential. **b** Changes of normalized coherence by DCZ injections. The ordinate is in a logarithmic scale. **c** Quantitative analysis of coherence differences between pre-DCZ and post-DCZ conditions. The ordinate is in a logarithmic scale. Areas enclosed by red lines indicate significant decreases (*P* < 0.01, flood-fill method). Areas enclosed by white lines indicate significant increases (*P* < 0.01, flood-fill method). **d** Proportion of channel pairs with significant Granger causality (GC). The abscissa is in a logarithmic scale. *n* = 11 sessions for each frequency bin. **e** Differences in GC in the MPFC-to-LH direction between post-DCZ and pre-DCZ conditions. The abscissa is in a logarithmic scale. Analysis period, 1–250 ms from stimulus onset. Data are mean ± SEM. Red circles, *P* < 0.05 (one-sided paired *t*-test). *n* = 11 sessions for each frequency bin. Source data are provided as a Source Data file.

To confirm that the target circuit was functionally affected, we performed electrophysiological recordings in MkP. First, we assessed inter-areal coordination, as indexed by coherence, using simultaneously recorded local field potentials (Fig. 3a). Although coherence increased phasically immediately after stimulus onset both before and after DCZ injections (Fig. 3b), the increase magnitude was significantly smaller after DCZ injections in the theta and alpha bands (Fig. 3c, blue areas enclosed by red lines; *P* < 0.01, subsampling). In the later half of the stimulus period, the coherence reduction remained significant in the partner-variable block (Fig. 3c, bottom), while enhancement was observed in the self-variable block (Fig. 3c, top, yellow areas enclosed by white lines). Second, we assessed the impacts of DCZ administrations on Granger causality from the MPFC to LH. The Granger causality test (see Methods) is a statistical hypothesis test to determine whether one time series (MPFC activity in the present study) is useful in predicting another (LH activity). During 1–250 ms after stimulus onset, substantial channel pairs exhibited significant Granger causality, particularly below the alpha band in both conditions (Fig. 3d). Importantly, Granger causality was significantly diminished after DCZ injections in the delta and theta bands in the partner-variable block (Fig. 3e; one-sided paired *t*-test, *P* < 0.05). These findings demonstrate that circuit-selective silencing impaired inter-areal coordination.

## Discussion

Using dual viral vectors combined with DREADD technology, we performed selective and reversible blockade of the circuit from the MPFC to LH. Our intervention protocol made animals significantly less susceptible to others’, but not one’s own, reward prospects. In line with the impact on the licking behavior, inter-areal coordination was diminished at the low-frequency bands after DCZ administrations. The presence of effects on social reward sensitivity suggests that the absence of effects on own reward sensitivity was not due to lack of action of the DREADD manipulation. The apparent lack of behavioral effects in the self-variable block could be explained, at least partly, by transient coherence enhancement and preserved information flow. It is conceivable that the transmission of self-reward information can be relayed and compensated by other brain regions, whereas other-reward information is transmitted specifically by the direct MPFC-to-LH circuit. The two kinds of signals carrying self-reward and other-reward information would eventually be integrated in the LH to compute a subjective reward value. This view is supported by our previous finding that the inactivation of LH neurons caused a significant decrease in sensitivity to both the self-reward probability and partner-reward probability^6^. An intriguing question for future work is whether this top-down circuit underlies complex social emotions, such as the sense of envy and inequality. In doing so, the monitoring of other ethological metrics, such as facial expressions, would also be useful for determining the neural substrates of social and emotional states^14^.

Our findings suggest that the MPFC-to-LH circuit conveys information about others’ reward prospects. Very recently, however, Tye and colleagues optogenetically activated MPFC neurons that projected directly to the LH while mice competed for rewards with a cage mate^15^. They found that animals undergoing optical stimulation won more rewards, had greater reward port occupations, and spent less time being displaced by competitors. The authors hypothesized that MPFC-to-LH circuits carry “social rank information” to achieve behavioral modulation during social competition. Then, how can their social rank hypothesis and our other-reward prospect hypothesis be reconciled? One possibility is that artificial activation of the MPFC-to-LH circuit may act to increase the prospect of others’ rewards, which in turn may drive one’s own dominance behavior to maintain social homeostasis^16^. On the other hand, social ranks and reward prospects are closely related aspects in social behavior, as evidenced by the example that dominant animals have priority access to food. Thus, what might appear as a discrepancy between the two hypotheses may not be fundamental. More importantly, the two studies may be interpreted from a different perspective. The behavioral tasks employed in Tye’s study and in ours differ in significant ways. In Tye’s task, direct physical contact and competition were allowed to obtain rewards, whereas in our task direct physical contact was not possible and real competition was not involved (reward outcomes were determined by a task-control computer). Thus, a win-lose contest was a crucial factor in Tye’s task, whereas other’s reward probability was essential information for reward valuation in our task. These task differences could explain divergent properties of neural activities and interpretations of behavioral alterations.

The circuit between the MPFC and amygdala has also been implicated in social reward processing^17^. Specifically, functional coordination between these two areas is concerned with decisions whether or not to deliver rewards to a conspecific^18^. Notably, prosocial decisions were associated with information flow from the amygdala to the ACCg, while selfish decisions were associated with information flow in the opposite direction^18,19^. These findings suggest that the MPFC-amygdala circuit is more specialized for determining other-regarding decision preferences depending on the social context at hand, whereas the MPFC-LH circuit may be more specialized for monitoring others’ reward availability and modulating dominance behavior under social competition. Further research is necessary to delineate the role for the circuit from the LH to the MPFC in social behavior.

## Methods

### Animals

We used three male macaques (*Macaca fuscata*; MkP, MkA, and MkD); MkP and MkA served as experimental subjects, and MkD as their partner. All animal care and experimental protocols were approved by the Institutional Animal Care and Use Committee of the National Institutes of Natural Sciences and carried out in accordance with the US National Institutes of Health *Guide for the Care and Use of Laboratory Animals*.

### Behavioral procedures

Two monkeys were placed in a social Pavlovian conditioning procedure^5^. Briefly, the two monkeys sat face-to-face across a horizontally placed monitor at a distance of 1.1 m from each other at eye level in a sound-shielded room. A trial was started when a visual stimulus (188 × 202 mm) was presented at the monitor center. One second later, the stimulus was switched off, and the reward feedback – either a water reward or nothing – was delivered to the partner (MkD) and, another 1 s later, to the self (MkP or MkA). When a water reward was given to the partner and the self, a low-pitched tone (125 Hz) and a high-pitched tone (1 kHz) were also presented, respectively. During data collection, the monkeys were not required to fixate any stimulus.

The social Pavlovian conditioning procedure was performed in two blocks of trials that differed in reward contexts: the self-variable block (120 trials) and the partner-variable block (120 trials). In the self-variable block, a set of three stimuli was used. Each stimulus was associated with self-reward at a different probability (*P* = 0.25, 0.5, or 0.75); however, all stimuli were associated with the same probability of partner’s reward (*P* = 0.2). In the partner-variable block, another set of three stimuli was used. Here, each stimulus was associated with partner’s reward at a different probability (*P* = 0.25, 0.5, or 0.75); however, all stimuli were associated with the same probability of self-reward (*P* = 0.2). The two blocks were alternated during data collection. The total amount of reward earned by each monkey was adjusted to be the same between the two blocks.

A reward constraint was incorporated into the task procedure to mimic resource limitation in the natural world. Specifically, both monkeys were never rewarded on the same trial. This means that the self could be rewarded only when the partner was not rewarded. Because of this constraint, the final outcome in each trial was either self-rewarded, partner-rewarded, or neither-rewarded. As described below, we focused on the stimulus period before any outcome was revealed. During the stimulus period, the probability of self-reward in the partner-variable block was objectively the same (*P* = 0.2) between different stimuli.

### Surgical procedure

The monkeys were anesthetized with ketamine HCl (10 mg/kg, i.m.) and xylazine (1–2 mg/kg, i.m.). General anesthesia was maintained with isoflurane (1–2%). After the skull bone was exposed, acrylic screws were installed to fasten the dental acrylic head implant under aseptic surgical conditions. A nonmetal head holder and recording chambers were stereotaxically installed and secured with dental acrylic. Craniotomy was performed under general anesthesia after the monkeys had been fully trained on the behavioral procedure. Antibiotics and analgesics were administered after surgery.

### Behavioral recording procedures

Licking movement was recorded using a vibration sensor (AE-9922; NF Corporation, Kanagawa, Japan) attached to a reward spout. Signals were amplified (60 dB), filtered (100–200 kHz), enveloped, and then digitalized at 1 kHz. Eye position was monitored using an infrared video tracking system at a time resolution of 500 Hz and a spatial resolution of 0.1° (iRecHS2; Human Informatics Research Institute, National Institute of Advanced Industrial Science and Technology). Water reward was delivered through a spout under the control of a solenoid valve, which was placed outside the sound-shielded room. Overt body movements were monitored continuously using a video-capturing system. Stimulus presentation, behavioral data collection, and reward delivery were controlled using a personal computer running the MATLAB MonkeyLogic toolbox (Jan 15, 2020, build 215)^20,21^.

### Neural recording procedures

Dual site recordings of LFPs were performed in the MPFC and LH using 16-channel electrodes (U/S probe; Plexon Inc., TX, USA). The inter-channel distance was 200 μm, and the impedance of each channel was 0.3–0.5 MΩ at 1 kHz. The electrodes were advanced using an oil-driven micromanipulator (MO-972A-D; Narishige, Tokyo, Japan). LFP signals were amplified, bandpass-filtered (0.2–300 Hz), and digitized at 1 kHz [PlexControl (ver. 1.20.0); OmniPlex Neural Recording Data Acquisition System; Plexon Inc., TX, USA].

### Identification of MPFC and LH

The MPFC and LH were identified electrophysiologically. The MPFC encompassed the rostral part of the pre-supplementary motor area (pre-SMA) and its further rostral extension within the medial wall, known as the prefrontal area 9m^22^. The pre-SMA of the macaque spans almost 5 mm in the rostro-caudal direction. The border between the pre-SMA and SMA was determined in accordance with physiological criteria^23^ on the basis of intracortical stimulation and somatosensory responses^5,24^. For the identification of the LH, the recording chamber was tilted 35° laterally in the coronal plane. When penetrations were directed toward the anterior division of the LH (0–2 mm posterior to the anterior commissure), electrodes typically passed through the globus pallidus, which was characterized by neurons with large-amplitude spikes and high-frequency discharges up to 100 Hz^25^, and then through a thin layer (almost 1 mm) of smaller-amplitude neurons with lower-frequency discharges, which was considered the substantia innominata^26^. When penetrations were directed toward the middle division of the LH (3–4 mm posterior to the anterior commissure), electrodes typically passed through the globus pallidus and fiber zones of the internal capsule. For penetrations directed toward the posterior division of the LH (5–6 mm posterior to the anterior commissure), electrodes passed through the thalamus, zona incerta, and fiber zones of the H field of Forel for more dorsal penetrations, and through the subthalamic nucleus and/or the substantia nigra pars reticulata for more ventral penetrations; their firing properties were consistent with previous studies^25,27,28^. In all divisions, neurons in the LH exhibited relatively low spontaneous firing rates (typically 5–10 Hz) and broad spike potentials. LH neurons frequently responded to the sight of food. The penetration tracks were histologically confirmed in MkA.

### Viral vector construction and injection

AAV vectors were prepared using the AAV Helper Free Expression System (Cell Biolabs, Inc., CA, USA)^29^. Briefly, the packaging plasmids (pAAV-DJ or rAAV2-retro helper [Addgene plasmid #81070] and pHelper) and transfer plasmid (pAAV-CAGGS-Cre or pAAV-EF1α-DIO-hM4D(Gi)-mCherry [Addgene plasmid #50461]) were transfected into HEK293T cells. AVV vector particles were purified by ultracentrifugation with cesium chloride. The purified particles were dialyzed with PBS containing 0.001% Pluronic F-68 (Sigma-Aldrich, MO, USA) and then concentrated using an Amicon 10 K MWCO filter (Merck Milli-pore, Darmstadt, Germany). The copy number of the viral genome (vg) was determined by real-time quantitative PCR using the TaqMan Universal Master Mix II (Applied Biosystems, CA, USA).

Vector injections were made bilaterally under pressure using a Hamilton microsyringe. For the LH, the AAV2-retro (7.5 × 10^12^ vg/mL) injections were made at two sites in each hemisphere with an inter-site distance of 2.0 mm in the rostro-caudal direction. The vectors were deposited at two different depths for each injection site, 0.8–1.0 mm apart from each other (0.5–0.75 μL at each depth). For the MPFC, the AAV-DJ (9.8 × 10^12^ vg/mL) injections were made at four sites in the rostro-caudal direction in each hemisphere, with an inter-site distance of 1.5 mm. The most caudal injection site was located 4.5 mm anteriorly to the SMA/pre-SMA border. The vectors were deposited at two different depths for each injection site, aiming at 3.0 mm and 5.0 mm from the cortical surface (0.5 μL at each depth).

### Vehicle and DCZ injection

Six weeks after the viral vector injections, we began the vehicle (control) and DCZ (test) experiments. The two conditions were alternately run on separate days. In the control condition, only saline (MkP) or 2% dimethyl sulfoxide (DMSO) with saline (MkA) was administered intramuscularly. In the test condition, DCZ (0.1 mg/kg) dissolved in 2% DMSO with saline was administered intramuscularly. Behavioral data collection started 10–15 min after vehicle or DCZ injections in accordance with previous studies^12,13^. In total, we performed the vehicle and DCZ experiment for 6 days (sessions) each in both MkP and MkA.

### Histology

MkA was deeply anesthetized and transcardially perfused with 0.1 M phosphate-buffered saline (PBS; pH 7.4) and then with 10% formalin in 0.1 M phosphate buffer (pH 7.4). The brain was removed from the skull and postfixed overnight. After saturation with 30% sucrose at 4 °C for 2 weeks, the brain was sectioned coronally at 50 μm thickness. A series of every tenth section was initially treated with 0.3% hydrogen peroxide in 0.1 M PBS for 30 min at room temperature to inhibit endogenous peroxidase. Subsequently, the sections were immersed in 1% skim milk for 1 h and incubated overnight at 4 °C with Living Colors DsRed Polyclonal Antibody (1:1000; Takara Bio USA, CA, USA) in 0.1 M PBS containing 0.1% Triton X-100 and 1% horse serum. The sections were incubated for 2 h in the same medium containing biotinylated horse anti-rabbit IgG antibody (1:500; Vector Laboratories, Peterborough, UK) and reacted with the ABC Elite kit (Vector Laboratories) for 1.5 h. For visualization of the antigen, the sections were reacted in 0.1 M PBS containing 0.02% 3,3′-diaminobenzidine, 0.01% Nickel, and 0.002% hydrogen peroxide. An adjacent series of sections was Nissl-stained with 5% Cresyl Violet.

### Statistics

No statistical methods were used to predetermine sample sizes; our sample sizes were, however, similar to those reported in previous studies (for example, see refs. ^13,30^). Data were assumed to be normally distributed, but this was not formally tested. Visual stimuli were presented pseudorandomly in each block. Data collection and analysis were not performed blinded to the conditions of the experiments. No data were excluded, except for (1) licking frequencies and licking modulations that were considered to be outliers based on median absolute deviations (MADs; threshold, 3 MAD) in MkA, (2) trials in which LFP values were considered to be outliers (threshold, 3 MAD), and (3) LFP data with problems of collinearity, nonstationarity, or heteroscedasticity for Granger causality analysis (see below). All statistical procedures were assessed by two-tailed tests, unless otherwise stated, and performed using commercial software [MATLAB 2018a (ver. 9.4) and 2020b (ver. 9.9) with Statistics and Machine Learning toolbox (ver. 11.4), Signal Processing toolbox (ver. 8.1), Parallel Computing toolbox (ver. 6.13), and Control System toolbox (ver. 10.5); MathWorks Inc., MA, USA].

### Data analysis

#### Licking movement

Anticipatory licking movements during the stimulus period were quantified as a behavioral measure of reward valuation. Each lick was detected as a discrete event using a threshold-crossing algorithm. The licking frequency during a 500-ms epoch in the stimulus period (MkP, 501–1000 ms after stimulus onset; MkA, 251–750 ms after stimulus onset) was quantified and z-score normalized using licking signals during a 500-ms epoch immediately before stimulus onset. Outliers were removed based on the MAD in MkA (threshold, 3 MAD). The relationship between the licking frequency and variable-reward probability was assessed using a Spearman rank correlation test (*P* < 0.05).

‘Licking modulation’ was quantified to assess the intervention effects. For each block in each experimental condition, all trials were grouped by variable-reward probabilities. Trials in each group were sorted in the chronological order. We paired the licking frequencies in the preferred trials with those in the non-preferred trials of the same chronological order. We defined the average of their differences (preferred minus non-preferred) as the ‘observed’ licking modulation. In the self-variable block, the preferred trials were those with a 75% chance of self-reward, and the nonpreferred trials were those with a 25% chance of self-reward. In the partner-variable block, the preferred trials were those with a 25% chance of partner-reward, and the non-preferred trials were those with a 75% chance of partner-reward. Outliers were excluded based on the MAD in MkA (threshold, 3 MAD). The observed licking modulation was compared between the vehicle and DCZ conditions (*P* < 0.05, Welch’s *t*-test).

We also employed a Monte Carlo permutation test to evaluate the significance of the difference in the licking modulation between the two experimental conditions. For this purpose, we created two simulated data sets. Specifically, licking frequencies were combined together across the two experimental conditions, separately for the preferred and nonpreferred trials. Suppose that there were 120 preferred trials and 120 nonpreferred trials in the vehicle condition, and 120 preferred trials and 120 nonpreferred trials in the DCZ condition. The combining procedure should yield 240 preferred trials and 240 nonpreferred trials. From these 240 preferred trials, we randomly selected 120 trials, and assigned them to a simulated vehicle condition and the remaining 120 trials to a simulated DCZ condition. Similarly, from 240 non-preferred trials, we randomly selected 120 trials, and assigned them to a simulated vehicle condition and the remaining 120 trials to a simulated DCZ condition. Using these simulated data sets, the ‘simulated’ licking modulation was computed in each condition in the same way as described above. Finally, a simulated intervention effect was computed by subtracting the licking modulation in the simulated DCZ condition from the licking modulation in the simulated vehicle condition. This shuffling procedure eliminated the effects of trials’ chronological order and experimental conditions under the null hypothesis of no intervention effect. This procedure was repeated 1000 times to obtain the distribution of simulated intervention effects. Finally, we examined whether the observed effect, i.e., the observed licking modulation in the vehicle condition minus the observed licking modulation in the DCZ condition, was above 97.5 percentile of the simulated distribution (*P* < 0.05, two-sided).

#### Field-field coherence

LFPs from all electrode channels (*n* = 16) were used in the MPFC, and those from selected channels were used in the LH. This was because several channels for LH recording were outside the LH, such as in the internal capsule and the globus pallidus. The LFPs were segmented from 2 s before to 2 s after stimulus onset for each trial. Trials in which the mean LFP value was considered to be an outlier were excluded (threshold, 3 MAD).

To compute field-field coherence between the MPFC and LH, the 4-s LFP segments were concatenated across trials into one long time series for each channel pair. The LFP signals were filtered to remove ham-related noise components around 60 Hz and 120 Hz. The filtered LFPs were processed at 24 frequency bins from 1 to 128 Hz by convolving with a complex Morlet wavelet function. The resulting coherence was divided into the original 4-s segments, averaged over trials, and normalized by subtracting the mean coherence 0–0.5 s before stimulus onset. The modulation of coherence was computed by subtracting the normalized coherence in the vehicle condition from those in the DCZ condition per channel pair (total *n* = 1,632 across recording sessions), which was then averaged in each block.

A subsampling hypothesis test was performed to determine significant regions of coherence modulation in the time-frequency plot. For this analysis, normalized coherence signals were concatenated along the contact-pair axis across the two experimental conditions, separately for the self-variable and partner-variable block. Consequently, for each block, a three-dimensional matrix was formed consisting of 1500 time points (i.e., from 500 ms before to 1000 ms after stimulus onset) × 24 frequencies × 3264 contact-pairs (i.e., 1632 × 2 conditions). We then randomly sampled 200 contact-pair data from the matrix, and randomly assigned half of them to a simulated vehicle condition and the remaining half to a simulated DCZ condition. The matrix for the simulated vehicle condition was subtracted from the matrix for the simulated DCZ condition to obtain simulated coherence modulation. This yielded a three-dimensional matrix consisting of 1500 time points × 24 frequencies × 100 contact-pairs. From a total of 3,600,000 data values within the matrix (i.e., 1500 × 24 × 100), the 0.5 percentile and the 99.5 percentile were chosen. This procedure was repeated 1,000 times to obtain two distributions. The distribution of the 0.5 percentile values was used to define a threshold for detecting regions in which significantly decreased coherence occurred; the distribution of the 99.5 percentile values was used to define a threshold for detecting regions in which significantly increased coherence occurred.

Specifically, if the observed value in any bin was below the 0.5 percentile point in the 0.5-percentile distribution, the bin was temporarily classified as a significant decrease. Likewise, if the observed value in any bin was above the 99.5 percentile point in the 99.5-percentile distribution, the bin was temporarily classified as a significant increase. A flood-fill algorithm was then applied to each candidate bin to identify significant clusters of bins. This method connected the candidate bins when their edges or corners touched along the horizontal, vertical, or diagonal direction. If a cluster consisted of less than 10 connected bins, it was removed. These thresholding and clustering procedures lowered the false-alarm rate in detecting coherence modulation in the time-frequency plot. Finally, the formed clusters were regarded as statistically significant regions of coherence modulation.

#### Granger causality

Granger causality from the MPFC to the LH was examined using outlier-removed LFPs as a measure of top-down information flow. The LFPs 1–250 ms after stimulus onset were analyzed using a multivariate linear vector autoregressive (MVAR) model^31^ provided by MATLAB Multivariate Granger Causality toolbox (ver. 1.0)^32^. The best MVAR model order up to 50 ms was determined using Akaike information criteria. The model parameters for the selected model order were estimated using ordinary least-squares regression. LFP time series data with problems of collinearity, nonstationarity, or heteroscedasticity were excluded. For the LFP data without the problems, the autocovariance sequence from the MVAR parameters was calculated. The pairwise conditional Granger causality was estimated using *F*-testing with a false discovery rate (*Q* < 0.05) at frequencies between 0 to 128 Hz with a 1.67-Hz step. The proportion of contact pairs with significant Granger causality from the MPFC to the LH was calculated at each estimated frequency in individual recording sessions (*n* = 11). A one-sided *t*-test was applied to determine whether the proportion at each estimated frequency was significantly lower in the DCZ condition than in the vehicle condition (*P* < 0.05).

### Reporting summary

Further information on research design is available in the Nature Portfolio Reporting Summary linked to this article.

## Supplementary information

Reporting summary

## Data Availability

All data are available within the paper and Source Data file. Source data are provided with this paper.

## Source data

Source Data

## Code availability

Behavioral and neural data analysis codes central to this paper are available at the Github repository (https://github.com/AtsushiNoritake/Noritake_etal_DCZexperiment) and published via Zenodo (10.5281/zenodo.8043230)^33^.
