# Supplementary material for: Chemogenetic dissection of a prefrontal-hypothalamic circuit for socially subjective reward valuation in macaques
Source: Nat Commun. 2023 Jul 20;14:4372. doi: 10.1038/s41467-023-40143-x (PMC10359292; doi:10.1038/s41467-023-40143-x)
Supplement: Supplementary file 1 — Reporting summary [file 41467_2023_40143_MOESM1_ESM.pdf]

nature portfolio

Corresponding author(s): Masaki Isoda

Last updated by author(s): Jun 20, 2023

Reporting Summary

Nature Portfolio wishes to improve the reproducibility of the work that we publish. This form provides structure for consistency and transparency in reporting. For further information on Nature Portfolio policies, see our Editorial Policies and the Editorial Policy Checklist.

Statistics

For all statistical analyses, confirm that the following items are present in the figure legend, table legend, main text, or Methods section.

n/a	Confirmed	
☐	☒	The exact sample size (*n*) for each experimental group/condition, given as a discrete number and unit of measurement
☐	☒	A statement on whether measurements were taken from distinct samples or whether the same sample was measured repeatedly
☐	☒	The statistical test(s) used AND whether they are one- or two-sided<br>*Only common tests should be described solely by name; describe more complex techniques in the Methods section.*
☐	☒	A description of all covariates tested
☐	☒	A description of any assumptions or corrections, such as tests of normality and adjustment for multiple comparisons
☐	☒	A full description of the statistical parameters including central tendency (e.g. means) or other basic estimates (e.g. regression coefficient) AND variation (e.g. standard deviation) or associated estimates of uncertainty (e.g. confidence intervals)
☐	☒	For null hypothesis testing, the test statistic (e.g. *F*, *t*, *r*) with confidence intervals, effect sizes, degrees of freedom and *P* value noted<br>*Give P values as exact values whenever suitable.*
☒	☐	For Bayesian analysis, information on the choice of priors and Markov chain Monte Carlo settings
☒	☐	For hierarchical and complex designs, identification of the appropriate level for tests and full reporting of outcomes
☐	☒	Estimates of effect sizes (e.g. Cohen's *d*, Pearson's *r*), indicating how they were calculated

Our web collection on statistics for biologists contains articles on many of the points above.

Software and code

Policy information about availability of computer code

Data collection	Behavioral and neural data were collected using MonkeyLogic toolbox (Jan 15, 2020 build 215) running on MATLAB 2018a (ver. 9.4) and Plexon OmniPlex Neural Recording Data Acquisition System software (PlexControl 1.20.0).
Data analysis	Data analysis was performed using Statistics and Machine Learning toolbox (ver. 11.4), Signal Processing toolbox (ver. 8.1), Parallel Computing toolbox (ver. 6.13), Control System toolbox (ver. 10.5), and Multivariate Granger Causality toolbox (ver. 1.0) provided by MATLAB 2018a (ver. 9.4) and 2020b (ver. 9.9) (Mathworks Inc., Natick, MA, U.S.A.). MATLAB codes central to this paper are available at the GitHub (https://github.com/AtsushiNoritake/Noritake_etal_DCZexperiment) and Zenodo (https://doi.org/10.5281/zenodo.8043230) repositories.

For manuscripts utilizing custom algorithms or software that are central to the research but not yet described in published literature, software must be made available to editors and reviewers. We strongly encourage code deposition in a community repository (e.g. GitHub). See the Nature Portfolio guidelines for submitting code & software for further information.

Data

Policy information about availability of data

All manuscripts must include a data availability statement. This statement should provide the following information, where applicable:

- Accession codes, unique identifiers, or web links for publicly available datasets
- A description of any restrictions on data availability
- For clinical datasets or third party data, please ensure that the statement adheres to our policy

All data are available within the paper and Source Data file. Source data are provided with this paper.

Human research participants

Policy information about studies involving human research participants and Sex and Gender in Research.

Reporting on sex and gender	N/A
Population characteristics	N/A
Recruitment	N/A
Ethics oversight	N/A

Note that full information on the approval of the study protocol must also be provided in the manuscript.

Field-specific reporting

Please select the one below that is the best fit for your research. If you are not sure, read the appropriate sections before making your selection.

☒ Life sciences ☐ Behavioural & social sciences ☐ Ecological, evolutionary & environmental sciences

For a reference copy of the document with all sections, see nature.com/documents/nr-reporting-summary-flat.pdf

Life sciences study design

All studies must disclose on these points even when the disclosure is negative.

Sample size	Two monkeys (MkA and MkP) participated in the study as experimental subjects (i.e., 'self'). Another monkey (MkD) participated in the study as a non-recorded partner. We collected behavioral data in the vehicle (n = 6 sessions) and DCZ (n = 6 sessions) conditions in both MkA and MkP. Neural data were collected from MkP (n = 11 sessions), where LFP activities were recorded from a total of 1632 channel pairs between the MPFC and LH. No statistical methods were used to predetermine these sample sizes, but our sample sizes were similar to those reported in previous publications (Hirabayashi et al., Neuron, 109, 3312-3322, 2021, Ref #13; Ninomiya et al. Nat Commun, 11, 5233, 2020, Ref #30).
Data exclusions	We excluded (1) licking frequencies and licking modulations that were considered to be outliers based on median absolute deviations (MADs; threshold, 3MAD) in MkA; (2) trials in which LFP values were considered to be outliers (threshold, 3MAD); and (3) LFP data with problems of collinearity, nonstationarity, or heteroscedasticity for Granger causality analysis (ref #32).
Replication	Two monkeys were used to verify the reproducibility of behavioral data and neural activity data in the vehicle and DCZ conditions. Reproducibility was confirmed.
Randomization	During the experiments, visual stimuli were presented pseudorandomly. In the procedures of the Monte Carlo permutation test and the subsampling test, randomization with a uniform distribution was performed.
Blinding	Data collection and analysis were not performed blinded to the conditions of the experiments because blinding was not possible due to the requirements of experimenter-controlled induction of protocols. However, the criteria used for data analyses were the same for different experimental conditions, and the analyses were performed automatically using MATLAB scripts.

Reporting for specific materials, systems and methods

We require information from authors about some types of materials, experimental systems and methods used in many studies. Here, indicate whether each material, system or method listed is relevant to your study. If you are not sure if a list item applies to your research, read the appropriate section before selecting a response.

March 2021

Materials & experimental systems

n/a	Involved in the study
☐	☒ Antibodies
☐	☒ Eukaryotic cell lines
☒	☐ Palaeontology and archaeology
☐	☒ Animals and other organisms
☒	☐ Clinical data
☒	☐ Dual use research of concern

Methods

n/a	Involved in the study
☒	☐ ChIP-seq
☒	☐ Flow cytometry
☒	☐ MRI-based neuroimaging

Antibodies

Antibodies used	Living Colors DsRed rabbit polyclonal antibody, Takara Bio USA. Inc., Catalog # 632496.<br>Biotinylated horse anti-rabbit IgG antibody, Vector Laboratories, Catalog # BA-1100.
Validation	For validation statements, relevant citations, and antibody profiles regarding the primary antibody, see:<br>https://www.takarabio.com/documents/Certificate%20of%20Analysis/632496/632496-101717.pdf

Eukaryotic cell lines

Policy information about cell lines and Sex and Gender in Research

Cell line source(s)	293T Cell (ATCC CRL-3216TM)
Authentication	Authenticated by ATCC
Mycoplasma contamination	Cell line was not tested for mycoplasma contamination.
Commonly misidentified lines<br>(See ICLAC register)	n/a

Animals and other research organisms

Policy information about studies involving animals; ARRIVE guidelines recommended for reporting animal research, and Sex and Gender in Research

Laboratory animals	Three macaque monkeys [Macaca fuscata; MkA (age 4), MkP (age 7), and MkD (age 11)] were used.
Wild animals	This study did not involve wild animals.
Reporting on sex	All animals were male.
Field-collected samples	This study did not involve samples collected from the field.
Ethics oversight	All animal care and experimental protocols were approved by the Institutional Animal Care and Use Committee of National Institutes of Natural Sciences.

Note that full information on the approval of the study protocol must also be provided in the manuscript.
